# RNA programming towards predictable control of living bacterial therapeutics

**DOI:** 10.1002/ctm2.70813

**Published:** 2026-08-31

**Authors:** Na Li, Shengkun Tong, Lijie Yin, Yaojie Gao, Yaojun Tong

**Affiliations:** ^1^ State Key Laboratory of Microbial Metabolism Shanghai Jiao Tong University Shanghai China; ^2^ Joint International Research Laboratory of Metabolic and Developmental Sciences Shanghai Jiao Tong University Shanghai China; ^3^ School of Life Sciences and Biotechnology Shanghai Jiao Tong University Shanghai China; ^4^ State Key Laboratory of Synergistic Chem‐Bio Synthesis Shanghai Jiao Tong University Shanghai China

1

Engineered bacteria are emerging as living medicines that reach diseased tissues, sense local cues and produce therapeutics in situ.^1^, ^2^ Yet their capacity to proliferate and adapt complicates therapeutic control: selective tumour colonisation does not guarantee selective treatment, and bacterial abundance and payload production can vary across lesions. The translational challenge is therefore to predict not only where therapeutic bacteria localise, but also when they activate and what local therapeutic exposure they generate.

Most current circuits address activation through transcriptional regulation. Tumour‐associated signals can be connected to inducible promoters, while externally applied energy provides an orthogonal means of controlling therapeutic expression.^3^ Quorum‐sensing modules can further provide population‐dependent activation once bacterial density reaches a critical threshold.^4^ In our ongoing development of an *Escherichia coli* Nissle 1917 platform, we are integrating tumour‐associated lactate sensing with an acyl‐homoserine‐lactone‐dependent population signal. The resulting AND gate is intended to initiate therapeutic expression only when both pathological and population criteria are satisfied, thereby separating physical localisation from functional activation.

Yet transcriptional logic alone does not determine therapeutic output. Promoter strength is commonly inferred from accumulated fluorescence under well‐mixed laboratory conditions, implicitly treating transcription, RNA stability, translation and protein accumulation as one process. In vivo, nutrient limitation, oxygen tension, pH, growth rate and host‐derived stress can all alter the relationship between promoter activity and final payload exposure. This becomes particularly important when a living therapeutic coordinates multiple effectors with distinct exposure‐response relationships, such as cytotoxic proteins, cytokines and metabolites.

Our recently developed SONAR (synthetic orthogonal nucleic acid activation and repression) system provides a programmable RNA‐control layer for addressing this gap.^5^ Attenuated Cas13d variants and rationally designed CRISPR RNAs enable transcript degradation, translation repression and IF3‐assisted translation activation using a single RNA‐guided effector. CRISPR RNA arrays further support compact, multiplexed regulation of individual open reading frames within polycistronic transcripts. In living therapeutics, transcriptional circuits could determine whether a program begins, while RNA‐level regulators tune its magnitude, duration and composition. Importantly, this architecture could decouple sensor calibration from payload calibration: once a disease‐sensing logic circuit is established, individual therapeutic outputs could be retuned through crRNA design without rebuilding the upstream sensing module. A potent cytokine could remain under a post‐transcriptional brake, whereas distinct guide RNAs could independently tune tumour‐killing, immune‐modulatory and metabolic payloads.

We therefore envision a multilayer sense‐decide‐tune‐treat framework. The chassis first reaches the pathological niche. Environmental and population signals determine whether activation is permitted. RNA programming calibrates execution, whereas payload‐release and safety modules govern therapeutic action and termination. This architecture therefore separates therapeutic decision from therapeutic execution: transcriptional logic determines whether bacteria act, whereas RNA programming determines how they act (Figure 1).

**FIGURE 1:**
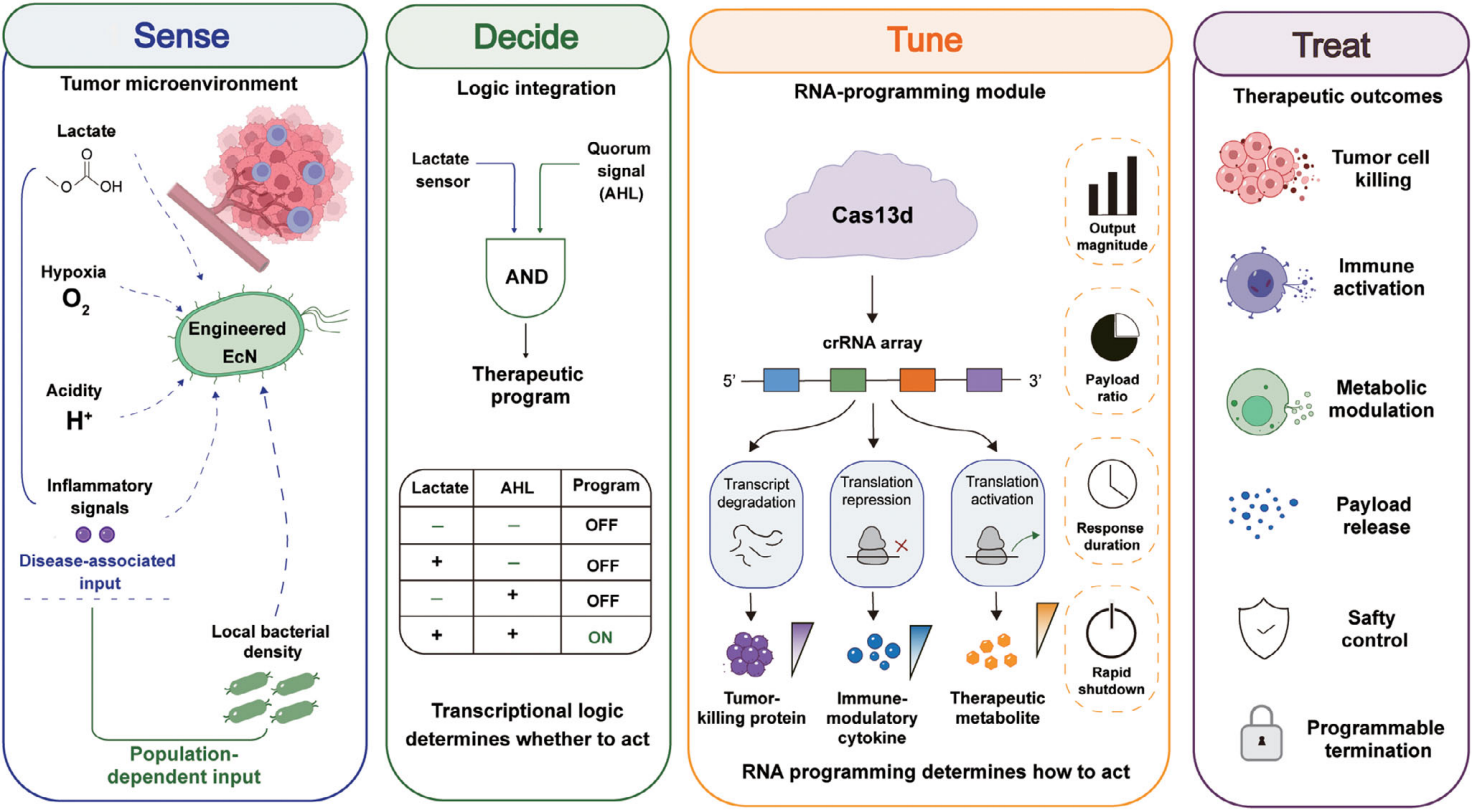
Multilayer programming of living bacterial therapeutics. Disease‐associated signals, exemplified by tumour lactate, are integrated with bacterial population signals through transcriptional logic to determine whether a therapeutic program should be activated. Programmable RNA regulators subsequently control transcript degradation, translation repression and translation activation, thereby tuning the magnitude, duration and composition of therapeutic outputs. Payload‐release and safety modules govern therapeutic action and termination. Transcriptional logic determines whether bacteria act, whereas RNA programming determines how they act.

The more fundamental opportunity is to establish a predictive relationship between in vitro circuit behaviour and in vivo therapeutic performance. For a living therapeutic, administered colony‐forming units (CFU) represent only the input dose, whereas pharmacologically relevant exposure is determined by the local amount of therapeutic molecules generated over time by active bacteria. Here, we use functional in vitro‐in vivo correlation to describe the mapping from environmental inputs and bacterial cellular states to local payload exposure. Conceptually, exposure depends on bacterial abundance, the fraction of cells in an active circuit state, per‐cell payload production, and subsequent payload release and clearance. A dose‐response curve measured at fixed optical density in rich medium is therefore insufficient if these relationships shift under physiologically relevant conditions.

This issue is especially important for tumour‐targeting bacteria. In vitro optimisation usually assumes homogeneous exposure to lactate or quorum signals, whereas tumours are spatially heterogeneous ecosystems. Lactate‐rich regions may not overlap with areas of highest bacterial density, and bacteria‐rich niches may not coincide with sites of full circuit activation. Even when bacteria are active, the spatial distribution of therapeutic exposure may diverge further because secretion, diffusion, stability and target binding are payload‐specific. Growth‐limited intratumoral bacteria may further alter promoter output, RNA stability and translational capacity. A circuit that appears robust in vitro may therefore fail in vivo because the mapping from environmental input to functional payload output has changed.

A predictive workflow should characterise circuit transfer functions and associated performance metrics, including threshold, dynamic range, kinetics, reversibility and fitness cost, under defined and physiologically relevant conditions. Spatial models such as tumour spheroids, organoids, microfluidic gradients and tissue slices can then test whether signal coincidence, bacterial distribution or RNA‐level tuning remains robust before animal studies (Figure 2).

**FIGURE 2:**
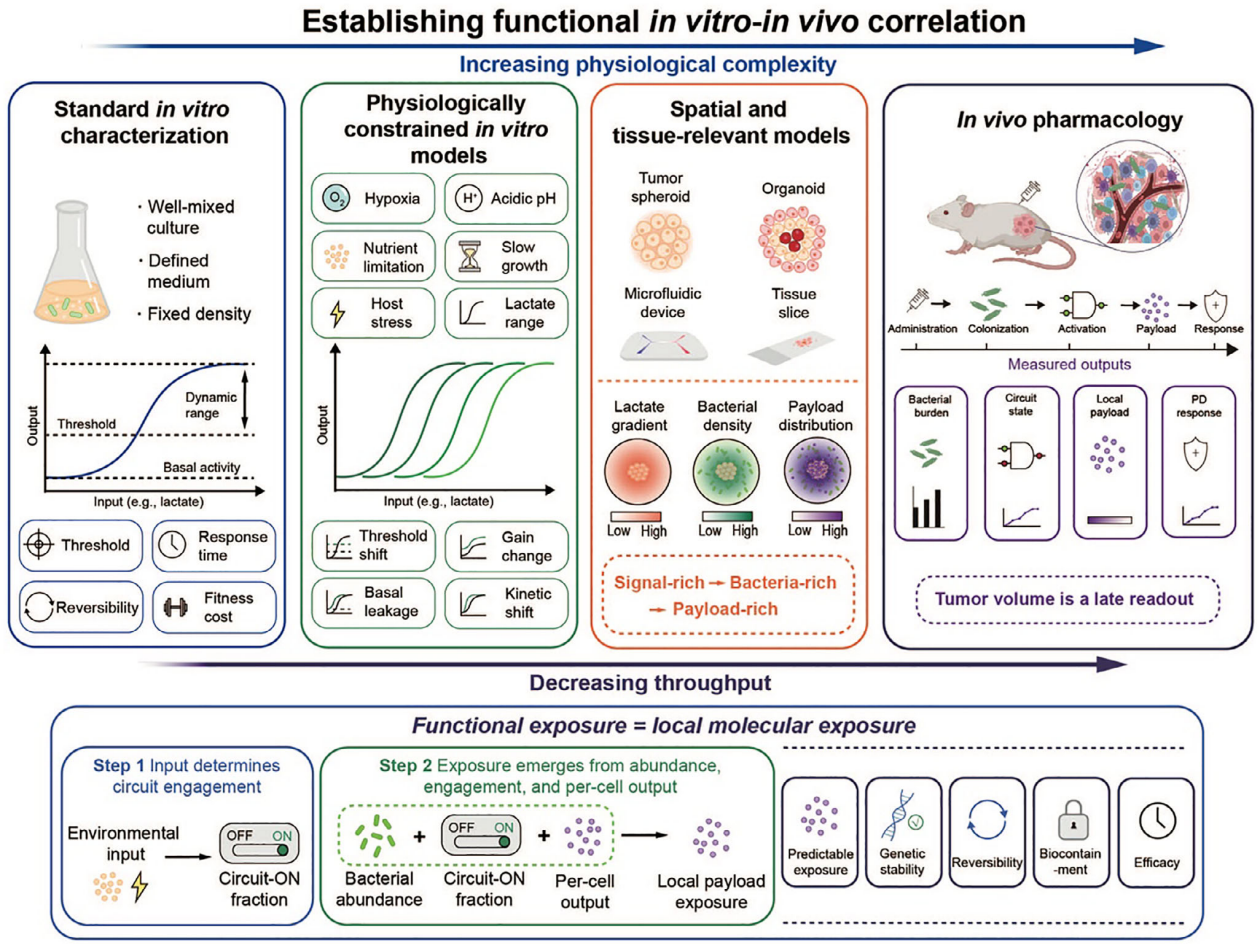
Establishing a functional in vitro‐in vivo correlation for living bacterial therapeutics. Circuit transfer functions are initially quantified under defined in vitro conditions and subsequently reassessed under physiologically constrained and spatially structured environments. Tumour spheroids, organoids, microfluidic gradients and tissue slices can reveal mismatches among environmental signals, bacterial distribution and payload production. In vivo studies should simultaneously quantify bacterial burden, circuit state, local payload exposure and pharmacodynamic response. Integration of these measurements establishes a functional in vitro‐in vivo correlation linking environmental inputs, bacterial abundance, circuit state and per‐cell output to local molecular exposure and pharmacodynamic response.

In vivo experiments should quantify bacterial burden, circuit state and intratissue payload concentration over time, rather than relying on tumour inhibition alone as evidence of circuit performance (Figure 2). Clinical studies of engineered EcN have already demonstrated that strain‐specific metabolites can provide dose‐responsive pharmacodynamic readouts of bacterial activity.^6^ Tumour volume is a delayed pharmacodynamic outcome that cannot distinguish insufficient colonisation from circuit inactivity, impaired translation, poor payload release or inadequate target engagement. Studies of programmable release, local checkpoint blockade delivery, bacterial encapsulation and quantitative live‐biotherapeutic development highlight the need to consider bacterial population dynamics together with payload exposure.^4^, ^7^, ^8^, ^9^ The same principle should apply to RNA‐programmed systems. Importantly, the relationship is bidirectional: payload production can alter bacterial fitness, whereas host responses can accelerate bacterial clearance, thereby feeding back on subsequent therapeutic exposure.

These dynamics also explain why apparently optimal constructs may fail in vivo. Prolonged basal expression can accumulate unwanted exposure, while high‐output circuits may favour non‐producing escape variants. Equivalent CFU may therefore generate different molecular exposures across distinct spatial and metabolic niches. Genetic stability and evolutionary robustness should consequently be evaluated together with circuit performance.^10^

For translational development, product characterisation should therefore extend beyond strain identity and administered CFU. A clinically meaningful potency assay should capture a state‐dependent input‐output function and, where feasible, connect it to local molecular exposure. Reporter fluorescence in vitro should ultimately be bridged to pharmacologically relevant outputs in vivo, including the concentration, duration and spatial distribution of active therapeutic molecules. Clinical precision will depend on whether these relationships remain predictable across physiological environments. Integrating transcriptional logic with programmable RNA regulation offers a route toward that goal.

## CONFLICT OF INTEREST STATEMENT

The author declares no conflict of interest.
